# Social determinants of older adult suicide: an ecological study using data of Japanese secondary medical areas

**DOI:** 10.1186/s12889-026-28241-z

**Published:** 2026-06-20

**Authors:** Shizuka Kudo, Tomoko Kodama, Yuri Sasaki

**Affiliations:** 1 Nomura Medical Clinic, 5942-1 Tsunehisa, Miyazaki City, Miyazaki Prefecture Japan; 2 National Institute of Public Health, 2-3-6 Minami, Wako City, Saitama Prefecture Japan

**Keywords:** Suicide, Older Adults, Gender differences, Ecological studies

## Abstract

**Background:**

Suicide prevention among older adults is a major public health concern in Japan, where the population is rapidly ageing. In 2020, approximately 40% of all suicide deaths occurred among people aged 60 years and older. Previous suicide prevention efforts have focused primarily on early detection and management of depression, while the social determinants of suicide among older adults remain underexplored. This study examined the associations between social indicators, aggregated lifestyle-related data derived from health checkups, and suicide rates among older adults to inform policy development.

**Methods:**

Using publicly available data, we examined the associations between the standardised mortality ratio (SMR) for suicide and socioeconomic factors, lifestyle, and lifestyle-related diseases among adults aged 60 years and older across secondary medical areas, which represent practical administrative units for implementing public health policies. Nonparametric correlation and multiple regression analyses were conducted.

**Results:**

Multiple regression analysis showed positive associations between suicide SMR and hypertension, smoking, daily alcohol consumption, older adult single-person households, and employment rate of older adults among men. The analysis showed negative associations with the number of psychotherapists and psychiatrists and income per taxpayer among women.

**Conclusions:**

Suicide risk among older adults demonstrates distinct gender-specific determinants. Preventive strategies should integrate health-related behaviours, socioeconomic factors, and mental health resource availability, and should be adapted to local contexts to effectively mitigate suicide risk, while acknowledging the limitations inherent in the ecological study design.

## Background

 In 2020, the number of suicide deaths among older adults aged 60 years and older in Japan was 8,126, accounting for approximately 40% of all suicide deaths [1]. Suicide prevention among older adults is an important issue in Japan, where population ageing is rapidly progressing. The suicide rate among older adults in Japan, as in most countries, exceeds that of other age groups, making it a global public health concern in ageing populations [2, 3].

Suicide among older adults is associated with mood disorders, including depression [4]. The combination of physical illness, isolation, loneliness, and depression in this population increases suicide risk [5, 6]. Previous suicide prevention efforts have tended to frame suicide as a personal problem, and measures were focused on the early detection and management of patients with depression and interventions for suicide attempters at high risk. However, such measures alone are insufficient; some researchers argue that greater attention should be paid to the social factors that contribute to the mental state ultimately leading individuals to commit suicide [7].

In recent years, increasing attention has been paid to the role of community- and area-level social determinants in mental health and suicide prevention among older adults. Recent studies have reported that neighbourhood socioeconomic disadvantage, low social cohesion, perceived neighbourhood disorder, and limited social connectedness are associated with depressive symptoms, hopelessness, and poor mental health outcomes among older adults [8–11].

Furthermore, studies from Europe and other countries have shown that area-level deprivation and social fragmentation are associated with increased suicide risk and suicide mortality among older adults [12–15]. For example, ecological studies in the United Kingdom and the Netherlands have demonstrated associations between neighbourhood socioeconomic deprivation, social fragmentation, and suicide rates [12, 13], while studies in Korea have reported that area-level socioeconomic disadvantage is associated with suicide mortality and suicidal ideation [14, 15].

In Japan, where rapid population ageing and regional demographic disparities are particularly pronounced, studies have additionally reported associations between suicide among older adults and rurality, regional social capital, socioeconomic deprivation, and social disconnection [16–19]. These findings suggest that both universal and country-specific social factors may contribute to suicide among older adults. Nevertheless, few studies have comprehensively examined the characteristics of secondary medical areas associated with suicide among older adults in Japan using multidimensional regional indicators. In addition, the association between mental health resources and suicide may vary depending on the socioeconomic context of the area; therefore, potential interaction effects between income and the availability of psychotherapists and psychiatrists warrant further investigation.

In Japan, the number of suicides among men is approximately twice that of women, and internationally, suicide mortality rates among men are consistently higher than those among women across most countries [20, 21]. Previous studies have suggested that gender differences in suicide may be influenced not only by biological factors but also by social and environmental factors, including employment status, social roles, social isolation, and help-seeking behaviour [22, 23]. Moreover, the influence of community and regional characteristics on suicide risk may differ by gender. Therefore, given the marked gender disparity in suicide rates, this study analysed regional characteristics associated with suicide among older adults separately by gender.

Additionally, to develop future suicide prevention measures, this study aimed to examine the association between area-level social, lifestyle, and healthcare-related factors and suicide mortality among older adults at the level of secondary medical areas in Japan. Specifically, we examined regional characteristics using indicators of social life [24–28], social participation among older adults [29], lifestyle factors and related diseases associated with mental symptoms such as depression [30–42], and healthcare delivery systems [43, 44], and identified the characteristics of areas with high suicide rates across secondary medical areas.

## Methods

### Research design

This ecological study used publicly available data from government statistics and other sources (Table 1). The association between factors related to suicide and the suicide standardised mortality ratio (hereafter ‘suicide SMR’) for people aged 60 years and older was analysed using nonparametric correlation analysis and multiple regression analysis by gender at the secondary medical area level—a unit suitable for policy implementation. In Japan, secondary medical areas are regional units established under the healthcare system for planning and coordinating general inpatient care and healthcare services at the regional level. These areas typically consist of multiple municipalities and are widely used for the allocation of healthcare resources and regional healthcare planning [45, 46]. In 2020, the mean population size of secondary medical areas was approximately 376,555 (range: 19,013–3,777,491). Among individuals aged ≥ 60 years, the mean population was 127,261 (range: 9,484–1,120,861) [47]. Accordingly, they represent practical administrative units for public health policy implementation and are suitable for examining area-level determinants of suicide among older adults.

**Table 1:** Data items and sources used in the analysis

Data items	Data sources	Years	Remarks
Number of suicide deaths by gender, 60 years and older	Suicide statistics (National Police Agency): Basic data on suicide in the region. Table A7. Residential area	2016-2020	Suicide Standardised Mortality Ratio SMR = D/Σpidi×100
			D: Average number of deaths in the target area in 2016–2020.
			pi: Population by five-year age group in the target area in 2020.
			di: Reference mortality ratio = (2016–2020, national average number of deaths in five-year age group)/(2020, national population by five-year age group).
Population, Population density, Proportion of single-person households aged 65 and older, Employment rate for those aged 65 and older	National Census	2020	
Income per taxpayer (thousands yen)	Chart of Municipal Taxation Status. Ministry of Internal Affairs and Communications.	2020	Total income (thousand yen)/Number of taxpayers for the per income levy.
			For Kawasaki City, the entire city’s data was used due to a lack of data from the northern and southern areas.
Total number of physicians, Number of psychotherapists and psychiatrists (both per 100,000 people aged 65 and older)	Statistics of Physicians, Dentists and Pharmacists. Ministry of Health, Labour and Welfare.	2020	
Number of *‘Kayoinoba’*, Number of participants in *‘Kayoinoba’* (per 100,000 people aged 65 years and older)	Survey on the implementation of comprehensive projects for disability prevention and daily life support (community support projects). Ministry of Health, Labour and Welfare.	2020	For Kawasaki City, the entire city’s data was used due to a lack of data from the northern and southern areas.
Lifestyle and lifestyle-related diseases: hypertension treatment, diabetes treatment, dyslipidaemia treatment, smoking, daily alcohol consumption, and exercise habits by gender, 40–74 years	The 8th National Database of Health Insurance Claims and Specific Health Checkups of Japan (NDB) Open Data. Specified Health Examination Questionnaire	2020	Question items 1, 2, 3, 8, 10, 18

### Data sources

The variables used in this study were derived from the following data sources. The number of suicide deaths was based on the National Police Agency’s suicide statistics (2016–2020: by 10-year age group). Data on community gathering places (*Kayoinoba*) were obtained from the Ministry of Health, Labour and Welfare’s survey on the implementation of comprehensive projects for disability prevention and daily life support (community support projects). Data on lifestyle and the prevalence of treatment for lifestyle-related diseases were obtained from the National Database of Health Insurance Claims and Specific Health Checkups of Japan (NDB) Open Data Specified Health Examination Questionnaire [48] (see Table 1).

As there were no published data of secondary medical areas other than the NDB open data, the data for each municipality were compiled for each secondary medical area (335 areas) based on the 2020 classification to construct the analytical database. Except for suicide deaths, data from 2020 was used.

### Variables

#### Dependent variables

As the suicide mortality rates fluctuate widely each year, the suicide SMR was calculated using the average number of suicide deaths among those aged 60 years and older over the five-year period of 2016–2020, and the expected number of suicide deaths among those aged 60 years and older using data from 2020.

$$\mathrm{SMR}=\mathrm D/{\textstyle\sum}\mathrm{pidi}\times100$$

D: Average number of deaths in the target area in 2016–2020.

Σpidi: Expected suicide deaths.

pi: Population by five-year age group in the target area in 2020.

di: Reference mortality rate = (2016–2020, national average number of deaths in five-year age group)/(2020 national population by five-year age group).

#### Explanatory variables

The following social life indicators were used: (1) population density, (2) older adult single-person households (defined as older adults living in single-person households and conceptually equivalent to ‘living alone’ in this study), (3) income per taxpayer (‘income’), as well as (4) total number of physicians and (5) number of psychotherapists and psychiatrists (both per 100,000 people aged 65 and older). For the indicators of social participation of older adults, (6) number of people participating in ‘*Kayoinoba*’ (7) and number of ‘*Kayoinoba*’ (both per 100,000 people aged 65 and older), as well as (8) employment rate of older adults (aged 65 and older) were used because public data for the municipalities and secondary medical regions across the country were scarce. The indices of lifestyle and lifestyle-related diseases were obtained from the NDB Open Data Specified Health Examination Questionnaire (conducted in 2020) [48], treatment of lifestyle-related diseases [(9) hypertension, (10) dyslipidaemia, (11) diabetes], (12) daily alcohol consumption, (13) smoking, and (14) exercise habits (two or more days per week) in the 40–74 age group were used.

### Analytical methods

Nonparametric correlation analysis was performed for the variables by gender, and Spearman’s rank correlation coefficient (ρ) was calculated. Moreover, multiple regression analysis was performed based on the following model for men and women using the suicide SMR as the dependent variable and the following explanatory variables: older adult single-person households, income, number of psychotherapists and psychiatrists, hypertension, diabetes, alcohol consumption, smoking, employment rate of older adults, and exercise habits. To examine whether the association between mental health resources and suicide varies by socioeconomic context, an interaction term between income and the number of psychotherapists and psychiatrists was included in the models. The employment rate of older adults, for which data were available separately for both men and women, was used as an indicator of social participation among older adults. In Models 3–5, lifestyle-related diseases (hypertension, diabetes) and lifestyle habits (smoking, alcohol consumption), which were correlated in the correlation analysis, and lifestyle habits (exercise habits), which were not correlated in the correlation analysis, were entered into the model in this order.

- Model 1: Older adult single-person households, income, and employment rate of older adults
- Model 2: Model 1 + number of psychotherapists and psychiatrists
- Model 3: Model 2 + hypertension and diabetes
- Model 4: Model 3 + drinking (daily) and smoking
- Model 5: Model 4 + exercise habits

In Models 2–5, a centralised interaction term between income and the number of psychotherapists and psychiatrists was added. Free-adjusted coefficients of determination were obtained for each model. To avoid multicollinearity between the explanatory variables, the variance inflation factor (VIF) was not greater than 10 in all the models.

Statistical analyses were conducted using the statistical software EZR version 1.62.

## Results

Descriptive statistics are shown in Table 2. The minimum and maximum suicide SMRs were 50.88 and 335.87 for men and 32.39 and 328.72 for women aged 60 years and older, respectively. Regarding lifestyle and lifestyle-related diseases, men scored higher than women in all categories except dyslipidaemia. Gender differences were observed, especially in diabetes, alcohol consumption, and smoking. The percentage of treated diabetes (mean ± SD) was 8.43 ± 1.27% among men and 4.15 ± 0.95% among women; smoking was 34.18 ± 3.19% among men and 9.76 ± 2.80% among women; and daily alcohol consumption was 39.81 ± 4.33% among men and 13.09 ± 2.33% among women.

**Table 2:** Descriptive statistics for each variable (*n* = 335)

	Minimum	Maximum	Mean	Standard deviation	Median (Interquartile Range)	
Suicide SMR aged 60 + Men	50.88	335.87	115.13	34.21	107.81	(93.40–129.89)
Suicide SMR aged 60 + Women	32.39	328.72	114.04	39.60	106.12	(90.35–130.83)
Population density (persons/km2)	12.49	18938.75	1195.16	2833.77	238.60	(87.21–674.95)
Older adult single-person households (%)	6.20	24.02	13.77	3.52	12.96	(11.11–16.08)
Income per taxpayers (thousand yen)	2335.36	6743.95	2937.41	441.66	2850.46	(2643.18–3122.20)
Employment rate aged 65+ (%) Men	26.92	51.21	36.70	4.12	36.08	(33.91–38.84)
Employment rate aged 65+ (%) Women	13.10	32.66	20.20	3.03	20.02	(17.78–21.80)
Number of *‘Kayoinoba’* (per 100,000 older adults aged 65+)	3.74	2487.91	403.04	294.55	347.79	(199.21–526.00)
Number of participants in *‘Kayoinoba’* (per 100,000 older adults aged 65+)	19.25	30850.09	6130.45	4221.57	5196.17	(2870.84–8250.30)
Total number of physicians (per 100,000 older adults aged 65+)	274.78	6284.43	717.03	434.06	615.91	(498.75–822.40)
Number of psychotherapists and psychiatrists (per 100,000 older adults aged 65+)	0.00	392.60	55.48	32.99	49.32	(37.07–70.07)
Hypertension 40ー74 years (%) Men	18.72	40.09	26.95	3.83	26.48	(24.10–29.42)
Hypertension 40ー74 years (%) Women	10.18	32.21	20.00	3.61	19.75	(17.69–22.09)
Dyslipidaemia 40ー74 years (%) Men	6.83	22.09	14.69	1.98	14.44	(13.47–15.85)
Dyslipidaemia 40ー74 years (%) Women	9.62	27.38	17.56	2.84	17.50	(15.52–19.06)
Diabetes 40ー74 years (%) Men	5.18	12.13	8.43	1.27	8.18	(7.50–9.28)
Diabetes 40ー74 years (%) Women	1.85	7.83	4.15	0.95	3.99	(3.51–4.64)
Smoking 40ー74 years (%) Men	25.18	42.34	34.18	3.19	34.03	(32.12–36.30)
Smoking 40ー74 years (%) Women	5.18	21.60	9.76	2.80	9.39	(7.82–10.80)
Daily alcohol consumption 40ー74 years (%) Men	27.93	52.97	39.81	4.33	39.17	(36.71–42.64)
Daily alcohol consumption 40ー74 years (%) Women	7.20	20.01	13.09	2.33	12.74	(11.57–14.59)
Exercise habits, at least 2 day/week 40ー74 years (%) Men	17.37	39.30	28.90	3.15	29.10	(26.78–30.95)
Exercise habits, at least 2 day/week 40ー74 years (%) Women	10.40	30.71	23.10	3.17	23.08	(20.92–25.29)

Correlation analysis showed that the suicide SMR among men was positively correlated with hypertension (ρ = 0.617), diabetes (ρ = 0.533), smoking (ρ = 0.499), older adult single-person households (ρ = 0.490), and alcohol consumption (ρ = 0.485) and negatively correlated with income (ρ = −0.564), population density (ρ = −0.533), total number of doctors (ρ = −0.459), and number of psychotherapists and psychiatrists (ρ = −0.318) (Table 3). For women, the suicide SMR was negatively correlated with population density (ρ = −0.353), total number of physicians (ρ = −0.344), and number of psychotherapists and psychiatrists (ρ = −0.307). No variables showed a positive correlation (ρ > 0.3), which was considered to indicate at least a moderate correlation based on conventional criteria [49] (Table 4).

**Table 3:** Spearman's rank correlation coefficients between each variable (men)

	Suicide SMR age 60+	Population density	Older adult single-person households	Income per taxpayers	Total number of physicians	Number of psychotherapists and psychiatrists	Number of participants in ‘Kayoinoba’	Number of ‘Kayoinoba’
Suicide SMR aged 60+	1	-	-	-	-	-	-	-
Population density	−0.533**	1	-	-	-	-	-	-
Older adult single-person households	0.490**	−0.630**	1	-	-	-	-	-
Income per taxpayers	−0.564**	0.757**	−0.638**	1	-	-	-	-
Total number of physicians	−0.459**	0.686**	−0.444**	0.565**	1	-	-	-
Number of psychotherapists and psychiatrists	−0.318**	0.512**	−0.281**	0.329**	0.719**	1	-	-
Number of participants in *‘Kayoinoba’*	0.219**	−0.240**	0.228**	−0.368**	−0.053	0.023	1	-
Number of *‘Kayoinoba’*	0.278**	−0.356**	0.296**	−0.463**	−0.147**	−0.063	0.946**	1
Employment rate	0.154**	−0.279**	−0.151**	−0.151**	−0.173**	−0.218**	0.026	0.056
Hypertension	0.617**	−0.677**	0.629**	−0.799**	−0.521**	−0.290**	0.265**	0.344**
Dyslipidaemia	0.248**	−0.411**	0.349**	−0.309**	−0.307**	−0.244**	0.049	0.0878
Diabetes	0.533**	−0.635**	0.559**	−0.687**	−0.509**	−0.290**	0.176**	0.243**
Smoking	0.485**	−0.487**	0.457**	−0.676**	−0.344**	−0.192**	0.350**	0.404**
Daily alcohol consumption	0.499**	−0.539**	0.308**	−0.549**	−0.542**	−0.322**	0.063	0.122 *
Exercise habits	−0.113 *	0.324**	0.097	0.170**	0.268**	0.274**	0.082	0.034

	Employment rate	Hypertension	Dyslipidaemia	Diabetes	Smoking	Daily alcohol consumption	Exercise habits
Suicide SMR aged 60+	-	-	-	-	-	-	-
Population density	-	-	-	-	-	-	-
Older adult single-person households	-	-	-	-	-	-	-
Income per taxpayers	-	-	-	-	-	-	-
Total number of physicians	-	-	-	-	-	-	-
Number of psychotherapists and psychiatrists	-	-	-	-	-	-	-
Number of participants in *‘Kayoinoba’*	-	-	-	-	-	-	-
Number of *‘Kayoinoba’*	-	-	-	-	-	-	-
Employment rate	1	-	-	-	-	-	-
Hypertension	0.080	1	-	-	-	-	-
Dyslipidaemia	0.025	0.534**	1	-	-	-	-
Diabetes	0.091	0.853**	0.569**	1	-	-	-
Smoking	0.168**	0.580**	0.087	0.391**	1	-	-
Daily alcohol consumption	0.138 *	0.550**	0.228**	0.597**	0.394**	1	-
Exercise habits	−0.326**	−0.032	−0.112 *	−0.109 *	−0.129 *	−0.394**	1

*p < 0.05, **p < 0.01

**Table 4:** Spearman's rank correlation coefficients between each variable (women)

	Suicide SMR age 60+	Population density	Older adult single-person households	Income per taxpayers	Total number of physicians	Number of psychotherapists and psychiatrists	Number of participants in *‘Kayoinoba’*	Number of *‘Kayoinoba’*
Suicide SMR age 60+	1	－	－	－	－	－	－	－
Population density	−0.353**	1	－	－	－	－	－	－
Older adult single-person households	0.132 *	−0.630**	1	－	－	－	－	－
Income per taxpayers	−0.253**	0.757**	−0.638**	1	－	－	－	－
Total number of physicians	−0.344**	0.686**	−0.444**	0.565**	1	－	－	－
Number of psychotherapists and psychiatrists	−0.307**	0.512**	−0.281**	0.329**	0.719**	1	－	－
Number of participants in *‘Kayoinoba’*	−0.017	−0.240**	0.228**	−0.368**	−0.053	0.023	1	－
Number of *‘Kayoinoba’*	0.037	−0.356**	0.296**	−0.463**	−0.147**	−0.063	0.946**	1
Employment rate	0.139 *	−0.243**	−0.088	−0.173**	−0.143**	−0.155**	0.070	0.094
Hypertension	0.259**	−0.660**	0.602**	−0.725**	−0.529**	−0.333**	0.216**	0.293**
Dyslipidaemia	0.250**	−0.576**	0.493**	−0.549**	−0.475**	−0.363**	0.182**	0.253**
Diabetes	0.193**	−0.587**	0.515**	−0.635**	−0.522**	−0.294**	0.139 *	0.211**
Smoking	−0.064	0.252**	−0.059	0.300**	0.257**	0.183**	−0.270**	−0.310**
Daily alcohol consumption	0.112 *	−0.052	0.089	0.017	−0.175**	−0.103	−0.293**	−0.301**
Exercise habits	−0.197**	0.358**	−0.020	0.286**	0.201**	0.210**	0.007	−0.054

	Employment rate	Hypertension	Dyslipidaemia	Diabetes	Smoking	Daily alcohol consumption	Exercise habits
Suicide SMR age 60+	－	－	－	－	－	－	－
Population density	－	－	－	－	－	－	－
Older adult single-person households	－	－	－	－	－	－	－
Income per taxpayers	－	－	－	－	－	－	－
Total number of physicians	－	－	－	－	－	－	－
Number of psychotherapists and psychiatrists	－	－	－	－	－	－	－
Number of participants in *‘Kayoinoba’*	－	－	－	－	－	－	－
Number of *‘Kayoinoba’*	－	－	－	－	－	－	－
Employment rate	1	－	－	－	－	－	－
Hypertension	0.080	1	－	－	－	－	－
Dyslipidaemia	0.073	0.756**	1	－	－	－	－
Diabetes	0.133 *	0.850**	0.680**	1	－	－	－
Smoking	−0.131	−0.300**	−0.315**	−0.376**	1	－	－
Daily alcohol consumption	−0.079	0.172**	−0.003	0.109 *	0.447**	1	－
Exercise habits	−0.203**	−0.082	−0.166**	−0.078	0.079	−0.031	1

*p < 0.05, **p < 0.01

Multiple regression analysis showed that the suicide SMR among men (Table 5) was positively correlated with older adult single-person households (β = 0.295, *p* < 0.01) and employment rate of older adults (β = 0.173, *p* < 0.01) and negatively correlated with income (β = −0.336, *p* < 0.01) in Model 2. In Model 3, the significant association with income identified in Model 2 disappeared, and hypertension (β = 0.416, *p* < 0.01), older adult single-person households (β = 0.171, *p* < 0.01), and employment rate of older adults (β = 0.139, *p* < 0.01) were positively associated. In Model 5, hypertension (β = 0.306, *p* < 0.01), smoking (β = 0.295, *p* < 0.01), alcohol consumption (β = 0.203, *p* < 0.01), older adult single-person households (β = 0.144, *p* < 0.05), and employment rate of older adults (β = 0.112, *p* < 0.05) were positively associated. The suicide SMR among women (Table 5) was positively associated with employment rate of older adults (β = 0.129, *p* < 0.05) and negatively associated with income (β = −0.247, *p* < 0.01) in Model 1. In Model 2, the significant association with employment rate identified in Model 1 disappeared, and income (β = −0.322, *p* < 0.01) and number of psychotherapists and psychiatrists (β = −0.262, *p* < 0.01) were negatively associated. In Model 4, income (β = −0.322, *p* < 0.01), number of psychotherapists and psychiatrists (β = −0.260, *p* < 0.01), and older adult single-person households (β = −0.151, *p* < 0.05) were negatively associated. In Model 5, the significant association with older adult single-person households identified in Model 4 disappeared, and income (β = −0.262, *p* < 0.05) and number of psychotherapists and psychiatrists (β = −0.245, *p* < 0.01) were negatively associated.

**Table 5:** Multiple regression analysis with suicide SMR as objective variable

Model	Variable	Standardised coefficient (β)		Standard error		95% confidence interval						Adjusted R-squared	
						Lower limit	Upper limit	Lower limit	Upper limit				
		men	women	men	women	men		women		men	women	men	women
1	Older adult single-person households	0.356	0.008	0.523	0.689	2.425	4.484	−1.265	1.445	**		0.293	0.073
	Income	−0.243	−0.247	0.004	0.005	−0.027	−0.011	−0.033	−0.011	**	**		
	Employment rate	0.224	0.129	0.386	0.691	1.104	2.623	0.329	3.047	**	*		
2	Older adult single-person households	0.295	−0.083	0.566	0.718	1.745	3.974	−2.349	0.476	**		0.303	0.124
	Income	−0.336	−0.322	0.005	0.007	−0.037	−0.016	−0.042	−0.015	**	**		
	Employment rate	0.173	0.061	0.418	0.706	0.611	2.256	−0.591	2.188	**			
	Number of psychotherapists and psychiatrists	−0.073	−0.262	0.063	0.080	−0.200	0.049	−0.472	−0.157		**		
	Income＊Number of psychotherapists and psychiatrists	0.182	0.292	0.000	0.000	0.000	0.000	0.000	0.000	**	**		
3	Older adult single-person households	0.171	−0.107	0.574	0.766	0.527	2.786	−2.709	0.307	**		0.371	0.123
	Income	−0.051	−0.267	0.006	0.008	−0.016	0.008	−0.040	−0.008		**		
	Employment rate	0.139	0.062	0.400	0.712	0.367	1.940	−0.583	2.216	**			
	Number of psychotherapists and psychiatrists	−0.095	−0.256	0.060	0.080	−0.217	0.020	−0.466	−0.150		**		
	Hypertension	0.416	0.127	0.813	1.237	2.113	5.311	−1.042	3.825	**			
	Diabetes	0.008	−0.044	2.179	4.212	−4.079	4.495	−10.105	6.466				
	Income＊Number of psychotherapists and psychiatrists	0.101	0.273	0.000	0.000	0.000	0.000	0.000	0.000		**		
4	Older adult single-person households	0.169	−0.151	0.565	0.804	0.528	2.749	−3.276	−0.113	**	*	0.426	0.128
	Income	0.151	−0.322	0.007	0.008	−0.001	0.025	−0.045	−0.012		**		
	Employment rate	0.104	0.055	0.393	0.719	0.093	1.637	−0.692	2.138	*			
	Number of psychotherapists and psychiatrists	−0.055	−0.260	0.059	0.081	−0.172	0.058	−0.472	−0.152		**		
	Hypertension	0.343	0.124	0.829	1.238	1.434	4.696	−1.070	3.800	**			
	Diabetes	−0.006	−0.007	2.291	4.332	−4.660	4.353	−8.823	8.222				
	Daily alcohol consumption	0.193	0.108	0.456	1.171	0.629	2.422	−0.472	4.135	**			
	Smoking	0.231	0.027	0.591	0.851	1.309	3.635	−1.288	2.060	**			
	Income＊Number of psychotherapists and psychiatrists	0.006	0.288	0.000	0.000	0.000	0.000	0.000	0.000		**		
5	Older adult single-person households	0.144	−0.128	0.584	0.826	0.253	2.549	−3.068	0.182	*		0.428	0.130
	Income	0.124	−0.262	0.007	0.009	−0.004	0.023	−0.042	−0.005		*		
	Employment rate	0.112	0.050	0.394	0.721	0.157	1.708	−0.769	2.066	*			
	Number of psychotherapists and psychiatrists	−0.075	−0.245	0.060	0.082	−0.195	0.040	−0.456	−0.132		**		
	Hypertension	0.306	0.154	0.854	1.261	1.056	4.416	−0.792	4.171	**			
	Diabetes	0.005	−0.010	2.293	4.328	−4.380	4.643	−8.913	8.116				
	Daily alcohol consumption	0.203	0.108	0.457	1.170	0.703	2.503	−0.471	4.132	**			
	Smoking	0.295	0.014	0.620	0.862	1.550	3.991	−1.494	1.897	**			
	Exercise	0.080	−0.078	0.560	0.744	−0.230	1.975	−2.434	0.494				
	Income＊Number of psychotherapists and psychiatrists	0.027	0.256	0.000	0.000	0.000	0.000	0.000	0.000		**		

*p < 0.05, **p < 0.01, Objective variable: Suicide SMR

## Discussion

The study suggested a positive association between the suicide SMR among older men and older adult single-person households, employment rate of older adults, hypertension, smoking, and daily alcohol consumption. Conversely, a negative association was found between the suicide SMR among older women and the number of psychotherapists and psychiatrists and income.

### Psychological well-being and gender differences

Loneliness and social isolation are frequently reported to have effects on mental health in addition to physical health. Loneliness is often reported to lead to deteriorating mental health, particularly among older adults [50]. Living alone is a risk factor for suicide due to loneliness regardless of gender and age, and its effect is greater on men than on women aged 70 years and older [51]. This study showed an association between older adult single-person households and the SMR among older men at the regional level.

A previous study on the association between social life indicators and the suicide SMR in healthcare areas in Iwate and Aomori prefectures reported an association between the suicide SMR among women and the number of physicians [28]. Another previous study reported a negative relationship between the presence of psychiatrists and suicide rates [44]. This study suggested an association between the suicide SMR among women and the number of psychotherapists and psychiatrists. Additionally, the interaction analysis suggests that the association between mental health resources and suicide may vary depending on the socioeconomic context of the area. Specifically, the availability of psychotherapists and psychiatrists may have different associations with suicide mortality across regions with varying income levels. This may partly explain the observed gender differences, particularly among women, although the underlying mechanisms remain unclear. Further research is needed to better understand how regional socioeconomic conditions modify the relationship between mental health service availability and suicide among older adults.

These findings may also reflect gender-specific mechanisms in how area-level factors influence suicide among older adults. In this study, such patterns were particularly observed among women, suggesting that area-level mental health resources may have a more context-dependent association with suicide in women than in men. For example, older men may be more affected by socioeconomic conditions such as employment and financial pressures, whereas older women may be more sensitive to the availability of social and healthcare resources, including access to mental health services. Differences in social roles, levels of social integration, and help-seeking behaviours between men and women may further contribute to these patterns. At the area level, such gender differences may be amplified by variations in community structure, resource distribution, and social support systems, highlighting the importance of considering gender-specific pathways in understanding regional disparities in suicide.

### Lifestyle habits and lifestyle-related diseases

Previous studies reported that lifestyle habits—such as exercise, smoking, and alcohol consumption— and lifestyle-related diseases—such as hypertension and diabetes—are closely related to mental health [30–42]. In particular, drinking [30] and smoking [33] are associated with suicide at the individual level among men in Japan. Previous cross-national studies using national-level aggregate data have not demonstrated an association between smoking and suicide among older adults [35], but this study suggests that smoking is associated with suicide among older men. Previous studies have reported an association between hypertension and depression at the individual level [41, 42], while the present study suggests an association with suicide. Although exercise and physical activity are useful in improving mental health [36, 37] and associated with suicide at the community level [52], this study did not find a significant association between exercise and physical activity and the suicide SMR. While some studies suggest that diabetes may increase the risk of depression and suicide, this study did not find an association between suicide SMR and diabetes [38–40]. The present study suggests that higher rates of smoking, alcohol consumption, and hypertension may be associated with higher suicide SMR among men at the community level. Therefore, efforts to prevent lifestyle-related diseases are important for preventing suicide among older adults, and coordinating suicide prevention with lifestyle-related disease prevention is crucial.

### Employment rate of older adults

Although this study found a positive association between the employment rate of older men and the suicide SMR, it is unclear whether a direct association exists because, rather than individual data, this study analysed population data. In areas where many older adults are employed for economic reasons, it is necessary to examine whether they are mentally trapped due to unimproved hardships or because employment itself creates stress factors. As population ageing progresses and the employment rate of older adults is expected to increase, the future employment environment for older adults should be closely examined.

### Limitations

This study analysed the suicide SMR and factors related to suicide among older adults in secondary medical areas. However, this study had some limitations. The analysis was insufficient due to the lack of published data in each secondary medical area, especially those related to older adults’ social participation. The variables related to ‘*Kayoinoba*’ (number of ‘*Kayoinoba*’ and number of participants), which are indicators of social participation among older adults, were not associated with the suicide SMR in Models 1–5 in the sub-analysis. Finally, as this was an ecological study, causality could not be stated, and ecological fallacies could not be ruled out.

## Conclusions

This study found gender differences in the relationship between suicide SMR among older adults and socioeconomic factors and lifestyle in secondary medical areas. A positive association was observed between older adult single-person households, employment rate of older adults, hypertension, smoking, and daily alcohol consumption among men. A negative association was found between the number of psychotherapists and psychiatrists and income among women. These findings underscore the importance of implementing comprehensive suicide prevention strategies that integrate health-related behaviours, socioeconomic factors, and mental health resource availability, while being tailored to local contexts and gender-specific differences.

## Data Availability

The source data underlying this study are publicly available.All data sources for this study are listed in Table 1 of this paper.
